# Factors associated with patient information sharing among home-visiting nurses in Japan: a cross-sectional study

**DOI:** 10.1186/s12913-019-3924-5

**Published:** 2019-02-04

**Authors:** Akiyo Nonogaki, Tomoko Nishida, Kazunari Kobayashi, Kayoko Nozaki, Haruka Tamura, Hisataka Sakakibara

**Affiliations:** 1 Department of Nursing, Graduate School of Medicine, Nagoya University, Institutional address: 1-1-20, Daiko-minami, Higashi-ku, Nagoya, Aichi 461-8673 Japan; 2 Department of Nursing, Sugiyama Jogakuen University, Institutional address: 17-3 Hoshigaoka-Motomachi, Chikusa-ku, Nagoya, Aichi 464-8662 Japan; 3 Nursing course, School of Medicine, Gifu University, Institutional address: 1-1, Yanagido, Gifu, Gifu 501-1194 Japan; 4 Home-visit nursing station Takayama, Institutional address: 588-1 Fuyutomachi, Takayama, Gifu, 506-0001 Japan

**Keywords:** Home health care agency, Home-visiting nurse, Information sharing, Job satisfaction, Patient information

## Abstract

**Background:**

Home-visiting nurses are expected to enhance their ability to provide adequate nursing care in a relatively isolated work environment. However, the isolated work environment leads to less opportunity to share patient information. We investigated factors relevant to better patient information sharing among home-visiting nurses, which would contribute to the improved care performance of these nurses.

**Methods:**

A cross-sectional study with anonymous self-administered questionnaire was conducted between June 2015 and September 2015 in two districts of Japan. Home-visiting nurses who were working at home health care agencies were recruited. The questionnaires consisted of items on demographic data, job-related variables, communication in the workplace, the current state of patient information sharing, opportunities (or measures) of patient information sharing in the workplace, and job satisfaction. Descriptive analyses were performed on all variables, using the Chi-square test, Fisher’s exact test, or Mann-Whitney U-test. Logistic regression analyses were conducted to identify the factors associated with better information sharing, adjusting the years of home-visiting nursing experience as the control variable.

**Results:**

Of 762 anonymous self-administered questionnaires were mailed, data from 482 participants who consented to this study and had no missing answer were analyzed. Of the total, 77.2% shared the patients’ information. Having a friendly adviser (OR = 2.51, 95% CI = 1.14–5.55, *p* = 0.023), attending some conferences (OR = 2.32, 95% CI = 1.12–4.82, *p* = 0.024), joining workshops (OR = 1.89, 95% CI = 1.15–3.10, *p* = 0.012), and years of home-visiting nursing experience (OR = 1.27, 95% CI = 1.03–1.57, *p* = 0.025) were significantly associated with sufficient sharing of the information. Nurses sufficiently sharing the information were well satisfied with their job (OR = 5.38, 95% CI =3.19–9.09, *p* < 0.001) and highly preferred a career in home-visiting nursing care (OR = 5.62, 95% CI =3.41–9.27, p < 0.001).

**Conclusions:**

The results suggested that having opportunities to discuss face-to-face such as at conferences and workshops as well as promoting good relationships among colleagues in the workplace will contribute to better information sharing among home-visiting nurses. Home-visiting nurses with less years of experience need to be supported in order to share the information sufficiently. Additionally, sufficient information sharing was also associated with job satisfaction and preference for home-visiting nursing care, which might lead to job retention for home-visiting nurses.

**Electronic supplementary material:**

The online version of this article (10.1186/s12913-019-3924-5) contains supplementary material, which is available to authorized users.

## Background

Access to patient information including active communication was the most important determinants of quality of care [1]. Lack of information can reduce home health provider’s ability to provide evidence-based disease management in the home [2]. Sufficient sharing of patient information reportedly contributed to understanding of patients, feeling at ease, and judgement with confidence [3]. In contrast to hospital nurses, who usually work as a group in a nurse station, home-visiting nurses generally work solely to visit a home patient with individual visiting schedule. Hence, they are unlikely to have enough time to share the patient information among colleagues or to receive frequent informal feedback from their superior [4]. Accordingly, the sharing of patients’ information relies on individual nurses and home health care agencies [5]. Insufficient communication among home-visiting nurses in an agency due to different visiting schedules may lead to lack of teamwork, less educational support, and a stressful work environment [6]. In contrast, good working environments, where nurses are valued, supported, and allowed to function fully and grow professionally, include open and informed communication throughout the organization, provision for staff to make choices, quick action and decision turnaround, and consistent validation of staff’s ideas [7].

Thus, better information sharing would help home-visiting nurses to reach higher performance levels in patient’s nursing care. However, there is no study investigating the factors for promoting information sharing among home-visiting nurses in the same agency, while the earlier studies mainly focused on hospital nurses or multi medical professionals.

We defined patient information as the essential information including nursing daily reports on patient care for close monitoring and appropriate intervention in maintaining patient wellness [8]. The information based on demographic information, prescriptions, and nursing observation. It consists of the patient’s condition reported by a home-visiting nurse colleague, physical information such as diseases and medical treatment, and living environment including family caregivers and utilization of medical care and social welfare services, which are necessary to provide individualized nursing care. Home-visiting nurses record care information of actual care operations as well as nursing reports, and share them with other nurses because they need to take care of a home-care patient by turns [9].

In addition, home visiting with confidence by sufficient information sharing may be linked to job satisfaction because nurses’ job satisfaction depends upon their sense of personal accomplishment [10]. Job satisfaction is an important factor in intent to leave the job, staff retention, and quality of care [11]. A theoretical model of job retention for home health care nurses proposed that job satisfaction is directly related to retention and indirectly related to retention though intent to stay [12]. The associated factors with job satisfaction among home-visiting nurses in Japan were reported hours of work, years of nursing experience, the number of home visiting, having a role of a care manager, joining workshops in the agency, having a conference, employment status, and having a supporter for housework [13]. These factors were estimated to be common with the factors associated with sufficient information sharing.

The first aim of this study was to investigate the situation of patient information sharing among home-visiting nurses in Japan and some factors relevant to better sharing of the information, which would contribute to the improved care performance of home-visiting nurses. The second aim was to clarify the relation between sharing the information and job satisfaction of home-visiting nurses.

## Methods

### Data and sample

A cross-sectional study design with anonymous self-administered questionnaire was used. Home-visiting nurses who were working at home health care agencies were recruited. Full-time employees and part-time employees were included. This study was carried out between June 2015 and September 2015 in two districts of Japan. The one located in urban area and another located in semi-urban and rural area in order to assess factors including different characteristics. Home health care agencies in the two districts were selected based on a database created by the national association for home-visiting nursing care. Most of all formal home health care agencies in Japan are registered in the database. Then, one of the authors telephoned all of the directors of the agencies on the database, which was totally 190 directors of agencies, in May and June 2015. One of the authors explained this study and asked them to receive the documents consisted of a letter of request, a written consent, a sample questionnaire, and an envelope, which explained the details of this study. The documents were mailed to 168 directors, and then 106 directors assented to participate in this study. They also informed us of the number of home-visiting nurses working at the agency. Based on the number, 762 anonymous self-administered questionnaires were mailed to 106 agencies. A total of 498 questionnaires were returned (response rate 65.4%). Of them, 482 were used for this analysis (valid response rate 63.3%). There were 16 incomplete questionnaires due to variables such as years of home-visiting nursing experience, information share-related variables, or job satisfaction measurement indices.

### Measures

We would investigate factors relevant to better sharing of the information and job satisfaction. The questionnaire was developed for this study (see Additional file 1). The factors were predicted personal and organizational characteristics, which include career, job condition, and agency size, and the opportunities and methods of information sharing for example nursing records, face-to-face communication, regular meeting, e-mail, and call, which were obtained from some earlier studies and my own experience as a home-visiting nurse [13–17]. Home-visiting nursing records are required legally, but no structural frameworks [18, 19]. The method of sharing of patients’ information relies on individuals’ and home health care agencies’ performance because there are no established system for sharing of the information [5]. Regular meetings and activities for staff will promote sharing, communication and support among the staff in terms of ideas, information and feelings [20]. However, it is reported that the frequency of regular meetings among home-visiting nurses are fewer than nurses working at hospitals [21]. These states might lead to difficulty of sufficient information sharing among home-visiting nurses, and then badly affect quality of care for a home patient.

#### Demographic variables

Demographic variables included sex, age, marital status, and having a child.

#### Job related variables

Job-related variables included items on the organization employing the nurses, their career history and licensures, and job conditions.

Questionnaire items on organization comprised agency size (small, < 5 nurses; medium, 5–9 nurses; large, > 9 nurses), the existence of clerks, being on call at night, and the night shift system.

Carrier and job conditions items covered license type, years of nursing experience, years of home-visiting nursing experience, hospital working experience, years of working at a current agency, employment status, pay system, the number of home visits per week, the number of days worked on the weekends/holidays per month, and the number of days of being on call at night per month.

#### Communication in the workplace

Communication in the workplace items covered eating lunch with colleagues in the last three months, having a friendly adviser in an agency, having a friendly director in an agency, and communication tools (“Do you use any communication tools to communicate with more than one nurse at the same time?”).

#### The current state of patient information sharing

The current state of patient’s information sharing item was assessed using one original item which asked “Can you visit a patient’s home with grasping patient’s information sufficiently?” The item was rated on a 4-point scale (1 = “Strongly agree,” 2 = “Agree,” 3 = “Disagree,” and 4 = “Strongly disagree”). Previous studies evaluated the state of information sharing by frequency of communication or self-evaluation [22–25]. In this study, the concept of sharing the patient information was defined as subjective awareness and one tool to gain self-confidence in nursing care. Therefore, the original item was used for self-evaluation for the current state of patient information sharing.

#### Opportunities or measures of patient information sharing in the workplace

Questionnaire items about opportunities or ways to share patient information consisted of the frequency of each instance in which the nurses used the opportunities (nursing record, talking with colleagues, e-mail, call, electronic chart, face-to-face hand over, summary of nurse report, workshop and conference). These items were rated on a 4-point scale (1 = “Always,” 2 = “Often,” 3 = “Rarely,” and 4 = “Never”).

#### Job satisfaction

Job satisfaction was measured with one original item of job satisfaction (“Are you satisfied with your current work?”) and items regarding high preference for a career in home-visiting nursing care, which were referred from the Nakano’s research paper (2008) of “The relation between retention and job satisfaction of visiting nurses.” These items were rated on a 4-point scale (1 = “Strongly agree,” 2 = “Agree,” 3 = “Disagree,” and 4 = “Strongly disagree”).

### Ethical considerations

Ethics committee approval was obtained from Graduate School of Medicine, Nagoya University, Japan (14–155).

### Data analysis

Descriptive analyses were performed on all variables, using the Chi-square test, Fisher’s exact test, or Mann-Whitney U-test. Logistic regression analyses were conducted, adjusting the years of home-visiting nursing experience as the control variable. The dependent variable was the situation of patient information sharing among home-visiting nurses, which were categorized into two groups based on whether a nurse can share patient information sufficiently or not. The group of sufficient information sharing consisted of the participants who answered, “Strongly agree” or “Agree.” In contrast, those who answered “Disagree” or “Strongly disagree” were categorized as the group of insufficient information sharing. Multivariable logistic regression models were further prepared to investigate the association of information sharing with some significant factors shown by logistic regression analyses and years of home-visiting nursing experience.

Indices of job satisfaction and high preference for a career in home-visiting nursing care were categorized into two groups of low or high by the median of the total score. Each item has from one to four points based on a 4-point scale. The total score of the three items of high preference for a career in home-visiting nursing care was calculated. Then, to investigate relation of these indices with the situation of patient information sharing, logistic regression analysis was conducted adjusting for the years of home-visiting nursing experience. The *p*-value of significance was set at 0.05 and significant trend was set at 0.1. The data were analyzed using IBM SPSS Statistics version 22.0 for Windows.

### Validity, reliability, and rigor

Cronbach’s alpha was measured to assess the reliability of high preference for a career in home-visiting nursing care items and was 0.861. The validity was assured by a Certified Nurse in Visiting Nursing and multiple researchers specialized in community health.

## Results

### Demographic characteristics

Table 1 shows sample demographic characteristics. The study participants ranged in age from twenties to sixties. They comprised 7 men (1.5%) and 471 women (98.5%). The most frequent years of nursing experience was in the range of 20 to 24 years (23.4%), while that of home-visiting nursing experience was less than 4 years (41.7%).

**Table 1:** Sample demographic characteristics (*n* = 482)

Variables	*n*	%
Sex		
Male	7	1.5%
Female	471	98.5%
Age		
< 29	13	2.8%
30–39	95	20.1%
40–49	208	44.1%
50<	156	33.1%
Agency size (The number of nurses)		
< 4	71	14.7%
5–9	192	39.8%
10<	219	45.4%
Years of nursing experience		
< 4	9	1.9%
5–9	50	10.5%
10–14	79	16.6%
15–19	97	20.4%
20–24	111	23.4%
25–29	81	17.1%
30<	48	10.1%
Years of home visiting nursing experience		
< 4	201	41.7%
5–9	130	27.0%
10–14	69	14.3%
15–19	61	12.7%
20–24	14	2.9%
25–29	6	1.2%
30<	1	0.2%
Having an experience of working at a hospital		
Yes	472	98.1%
No	9	1.9%
Employment status		
Full-time	297	61.7%
Part-time	184	38.3%
Pay system		
Monthly-pay system	298	62.3%
Hourly-pay system	92	19.2%
Fixed by the number of home visiting	88	18.4%
The number of home visiting (/week)		
1–19	283	59.3%
20–29	163	34.2%
30<	31	6.5%
The number of days being on call at night (/month)		
0	178	37.2%
1–4	138	28.8%
5<	163	34.0%

### Demographic and job related characteristics associated with sharing information

As shown in Table 2, sufficient sharing of patient information among colleagues was associated with age (*p* = 0.001), and years of home-visiting nursing experience (*p* = 0.003). The years of home-visiting nursing experience was correlated with age (Spearman’s rho = 0.407).

**Table 2:** Demographic and job related characteristics associated with sharing information (n=482)

	Sharing information				
	No		Yes		
Variables	*n*	%	n	%	*P*
Age^†^					
< 29	7	53.8%	6	46.2%	0.001
30–39	23	24.2%	72	75.8%	
40–49	58	27.9%	150	72.1%	
50<	20	12.8%	136	87.2%	
Agency size (The number of nurses)^†^					
< 4	22	31.0%	49	69.0%	0.302
5–9	39	20.3%	153	79.7%	
10<	49	22.4%	170	77.6%	
Existence of clerks^‡^					
Yes	93	22.5%	320	77.5%	0.652
No	17	25.0%	51	75.0%	
Having an experience of working at a hospital^‡^					
Yes	106	22.5%	366	77.5%	0.440
No	3	33.3%	6	66.7%	
Years of nursing experience^†^					
< 9	19	32.2%	40	67.8%	0.191
10–19	39	22.2%	137	77.8%	
20<	51	21.3%	189	78.8%	
Years of home-visiting nursing experience^†^					
< 9	88	26.6%	243	73.4%	0.003
10–19	20	15.4%	110	84.6%	
20<	2	9.5%	19	90.5%	
Employment status^‡^					
Full-time	62	20.9%	235	79.1%	0.235
Part-time	47	25.5%	137	74.5%	
Pay system^‡^					
Monthly-pay system	61	20.5%	237	79.5%	0.840
Hourly-pay system	32	34.8%	60	65.2%	
The number of home visiting	15	17.0%	73	83.0%	
The number of home visiting (/week)^†^					
1–19	69	24.4%	214	75.6%	0.147
20–29	37	22.7%	126	77.3%	
30<	2	6.5%	29	93.5%	
The number of days working on weekends/holidays (/month)^†^					
0	35	23.3%	115	76.7%	0.957
1–2	43	20.9%	163	79.1%	
3<	29	24.0%	92	76.0%	
Talking with colleagues^‡^					
No	4	57.1%	3	42.9%	0.049
Yes	106	22.3%	369	77.7%	

^†^Mann-Whitney U-test ^‡^Chi-square test/Fisher’s exact test

### Characteristics associated with sharing information

Table 3 shows characteristics associated with sharing information using a logistic regression model. Only the years of home-visiting nursing experience was adjusted because the years of nursing experience was thought to have greater effects on the ability of information sharing compared to age as a biological factor. Nurses who could share the information sufficiently had a greater number of days being on call at night (OR = 1.80, 95% CI = 1.07–3.03, *p* = 0.027), joining workshops in/out of the agency (OR = 2.47, 95% CI = 1.57–3.88, *p* < 0.001), eating lunch with colleagues (OR = 1.63, 95% CI = 1.00–2.67, *p* = 0.049), having a friendly adviser in the agency (OR = 2.63, 95% CI = 1.25–5.55, *p* = 0.011), and having a friendly director (OR = 3.86, 95% CI = 2.07–7.21, *p* < 0.001). The analysis of having a friendly director was conducted, excluding 61 directors.

**Table 3:** Characteristics associated with sharing information (*n* = 482)

	Sharing information						
	No		Yes				
Variables	*n*	%	*n*	%	OR	95%CI	*P*
The number of being on call at night(/month)							
0	52	29.2%	126	70.8%	1.00	(ref)	
1–4	28	20.3%	110	79.7%	1.41	0.82–2.42	0.210
5<	29	17.8%	134	82.2%	1.80	1.07–3.03	0.027
Joining workshops in/out of the agency							
No	50	35.7%	90	64.3%	1.00	(ref)	
Yes	58	17.1%	281	82.9%	2.47	1.57–3.88	< 0.001
Eating lunch with colleagues							
No	32	29.6%	76	70.4%	1.00	(ref)	
Yes	77	20.8%	294	79.2%	1.63	1.00–2.67	0.049
Having a friendly adviser in the agency							
No	14	43.8%	18	56.3%	1.00	(ref)	
Yes	94	21.0%	353	79.0%	2.63	1.25–5.55	0.011
Communicating tools							
No	56	25.0%	168	75.0%	1.00	(ref)	
Yes	54	20.9%	204	79.1%	1.31	0.85–2.01	0.226
Having a friendly director(※*n* = 421)							
No	25	51.0%	24	49.0%	1.00	(ref)	
Yes	74	19.9%	298	80.1%	3.86	2.07–7.21	< 0.001
Nursing records							
No	5	20.0%	20	80.0%	1.00	(ref)	
Yes	105	23.1%	350	76.9%	0.94	0.34–2.60	0.942
E-mail							
No	70	26.3%	196	73.7%	1.00	(ref)	
Yes	40	18.5%	176	81.5%	1.47	0.94–2.29	0.091
Call							
No	55	26.3%	154	73.7%	1.00	(ref)	
Yes	55	20.1%	218	79.9%	1.27	0.82–1.96	0.285
Summary of nurse record							
No	15	27.3%	40	72.7%	1.00	(ref)	
Yes	95	22.4%	330	77.6%	1.2	0.63–2.30	0.567
Electronic chart							
No	90	24.0%	285	76.0%	1.00	(ref)	
Yes	20	19.2%	84	80.8%	1.21	0.70–2.10	0.491
Hand over							
No	31	30.4%	71	69.6%	1.00	(ref)	
Yes	79	20.8%	300	79.2%	1.75	1.07–2.88	0.027
Conference in the agency							
No	29	46.0%	34	54.0%	1.00	(ref)	
Yes	81	19.4%	336	80.6%	3.25	1.86–5.69	< 0.001

Logistic regression analysis after adjustment for the years of home-visiting nursing experience

※Except directors

Concerning opportunities or measures of information sharing, sufficiently sharing the information was associated with having opportunities to hand over the information (OR = 1.75, 95% CI =1.07–2.88, *p* = 0.027), and to attend some conferences in the agency (OR = 3.25, 95% CI = 1.86–5.69, *p* < 0.001). Although talking with colleagues was also found as a significant factor, only 1.5% of persons did not talk with colleagues. Nurses using e-mails tended to share the information sufficiently (OR = 1.47, 95% CI = 0.94–2.29, *p* = 0.091).

### Factors associated with sufficient sharing of the information

Table 4 presents the results of the multiple regression analysis. Having a friendly adviser (OR = 2.51, 95% CI = 1.14–5.55, *p* = 0.023), attending some conferences in the agency (OR = 2.32, 95% CI = 1.12–4.82, *p* = 0.024), joining workshops in/out of the agency (OR = 1.89, 95% CI = 1.15–3.10, *p* = 0.012), and years of home-visiting nursing experience (OR = 1.27, 95% CI = 1.03–1.57, *p* = 0.025) were significantly associated with sufficient sharing of the information.

**Table 4:** Factors associated with sufficient sharing information (*n* = 482)

Variables	OR	95%CI	*P*
Years of home visiting nursing experience	1.27	1.03–1.57	0.025
The number of being on call at night	1.20	0.90–1.59	0.213
Joining workshops in/out of the agency	1.89	1.15–3.10	0.012
Eating lunch with colleagues	1.14	0.65–1.99	0.653
Having a friendly adviser in the agency	2.51	1.14–5.55	0.023
Attending hand over	0.79	0.47–1.78	0.912
Attending some conferences in the agency	2.32	1.12–4.82	0.024

Forced entry multiple regression analysis

Dependent variables: Sharing information

Independent variables: Years of home visiting nursing experience, The number of being on call at night, Joining workshops in/out of the agency, Eating lunch with colleagues, Having a friendly adviser in the agency, Attending hand over, Attending some conferences in the agency

### Job satisfaction associated with sharing information

Job satisfaction was also associated with sufficient sharing of the information (Table 5). Nurses sufficiently sharing the information were well satisfied with their job (OR = 5.38, 95% CI =3.19–9.09, *p* < 0.001) and highly preferred a career in home-visiting nursing care (OR = 5.62, 95% CI =3.41–9.27, *p* < 0.001).

**Table 5:** Job satisfaction associated with sharing information (n = 482)

	Sharing information						
	No		Yes				
Variables	*n*	%	*n*	%	OR	95%CI	*P*
Satisfied with my job							
No	41	53.2%	36	46.8%	1.00	(ref)	
Yes	69	17.0%	336	83.0%	5.38	3.19–9.09	< 0.001
Preference for home-visit nursing care							
Low	48	53.3%	42	46.7%	1.00	(ref)	
High	62	15.8%	330	84.2%	5.62	3.41–9.27	< 0.001

Logistic regression analysis after adjustment for the years of home-visiting nursing experience

## Discussion

In this study, the association between sufficient sharing of the information among home-visiting nurses and the relevant factors was investigated for the first time.

The present results showed that having a friendly adviser, attending some conferences in the agency, and joining workshops in/out of the agency were associated with sufficient sharing of the information among home-visiting nurses. Hospital nurses generally work together in the hospital, so that they are thought to have more opportunities such as having a friendly adviser in workplace, attending some conferences, and joining workshops. However, because home-visiting nurses mostly work at the patients’ homes alone, they are likely to lack opportunities to talk with colleagues directly and create good relationships among them. They also reportedly have little opportunities of getting advice and evaluation on nursing care from others [26, 27]. Good communication and advice networks of nursing staff promote the high level of cooperation that is needed to provide good care [22]. Having a friendly adviser in an agency can help not only sharing patients’ information, but also exchanging opinions on patient care. Attending some conferences or joining workshops can also provide home-visiting nurses with opportunities to exchange and share their opinions and experiences on patient care face-to-face. Thus, these face-to-face opportunities will contribute to sufficient sharing of patient information beyond the nursing record and probably better patient care as well.

Home health care agencies in Japan are typically small organizations, leading to a lack of meetings and conferences or poor relationships among the staff [28]. Home-visiting nurses sometimes visit their patients’ homes directly without stopping by their agencies. It is, therefore, difficult for them to have time enough to attend conferences or workshops to share patient information. The present findings suggest that having a friendly adviser and opportunities for face-to-face discussion such as attending some conferences and some workshops will develop a good relationship among colleagues and advice networks in workplace. It is, hence, considered that providing such face-to-face opportunities can be effective in sufficient sharing of the information and also better nursing care.

More years of home-visiting nursing experience was verified as one of the factors associated with sufficient sharing of the information. This is because home-visiting nurses with more experience may have abilities to provide adequate care with sufficient patient information sharing. More years of working experience in health care or experience in the current ward was linked with positive views about the support of individuality [29]. Experienced nurses become more comfortable and confident in their own practice because they are able to extend their interest from the merely technical aspects of care provision to the broader features of the care environment and individuals [30]. Thus, nurses with more experience may more easily understand what patient information is essential and required to provide adequate nursing care with confidence. On the contrary, this study suggests that home-visiting nurses with less years of experience need to be supported in sharing the essential information so as to understand the patient’s situation.

In this study, 41.7% of respondents had less than 4 years of working experience as home-visiting nurses. However, regarding working experience as a nurse, many had the experience of 20 to 24 years. According to the data of the Japanese Nursing Association, 70.0% of home-visiting nurses changed their jobs from hospitals or clinics to home health care agencies [31]. Namely, in the current Japanese situation, a large number of home-visiting nurses are inexperienced though they are much experienced at hospitals or clinics. This study showed that less years of home-visiting nursing experience tended to be linked with insufficient sharing of patient’s information, which may lead to insufficient understanding of the patient’s situation. Learners acquire knowledge not solely from education programs or books, but also through daily practice [32]. Therefore, educational supports in daily practice for inexperienced home-visiting nurses including novice nurses are essential so as to enhance quantity and quality of home-visiting nursing care. Having a friendly adviser and opportunities to attend conferences and workshops will contribute to such supports for inexperienced nurses as well.

This study also indicated that nurses sufficiently sharing the information were likely to be satisfied with their job and highly preferred a career in home-visiting nursing care. Job tenure and job satisfaction were the strongest predictors of nurse retention [7]. One of the factors influencing home care nurse intention to remain employed was supportive work relationship [33]. Education for novice nurses and continuation of conferences and discussion could produce novice nurses’ confidence in practice, and so their intention of remaining employed [34]. Consequently, this study suggests that good relationships or discussion among colleagues should be considered to promote sufficient sharing of the information and also high job satisfaction, and then may lead to job retention as a home-visiting nurse.

### Study limitations

One of the limitations of this study was that sufficient sharing of the information was defined based on only one original questionnaire item of whether nurses can share the information sufficiently or not. More comprehensive assessments of sufficient sharing would be desirable for further study. Second, the participants in this study might be better at information sharing or had high job satisfaction to begin with because the reason of participation in this study will be an interest in information sharing or job satisfaction. Third, the participants were limited because home-visiting nurses who worked at home healthcare agencies in only two districts of Japan were selected. Finally, the cross-sectional design of this study does not imply cause-effect conclusions regarding information sharing among home-visiting nurses and job satisfaction.

## Conclusion

Home-visiting nurses have some difficulties in sharing patients’ information among colleagues due to their isolated work environment. This study suggested that better information sharing among home-visiting nurses could be improved by having a friendly adviser and having opportunities to discuss with each other face-to-face such as at conferences and workshops. Home-visiting nurses with less years of experience need to be supported in order to share the information sufficiently. Additionally, sufficient information sharing was also associated with job satisfaction and preference for home-visiting nursing care, which might lead to job retention for home-visiting nurses.

## Additional file

Additional file 1: The questionnaire (English version). The original Japanese version of the questionnaire was translated into English as a supplemental information. (DOCX 28 kb)
