# Supplementary material for: Factors associated with patient information sharing among home-visiting nurses in Japan: a cross-sectional study
Source: BMC Health Serv Res. 2019 Feb 4;19:96. doi: 10.1186/s12913-019-3924-5 (PMC6360686; doi:10.1186/s12913-019-3924-5)
Supplement: Supplementary file 1 — The questionnaire (English version). The original Japanese version of the questionnaire was translated into English as a supplemental information. (DOCX 28 kb) [file 12913_2019_3924_MOESM1_ESM.docx]

**＜Questionnaire of patient information sharing among home-visiting nurses＞**

**Your personal characteristics, job condition and patient information sharing are asked.**

**Please circle the number of each question.**

| Sex | 1．Male | 2．Female |  |  |  |
| --- | --- | --- | --- | --- | --- |
| Age | **1．<24** | **2．25-29** | **3．30-34** | **4．35-39** | **5．40-44** |
|  | **6．45-49** | **7．50-54** | **8．55-59** | **9．60<** |  |
| Partner | **1．Have** | **2．No have** |  |  |  |
| Child | **1．Have** | **2．No have** |  |  |  |

1. About your working agency.

①How many home-visiting nurses work at the agency?

| 1．<5 | 2．5-9 | 3．10< |
| --- | --- | --- |

②Does an office clerk work at the agency?

| 1．Yes | 2．No |
| --- | --- |

③Does the agency provide 24-hour service?

| 1．Yes | 2．No |
| --- | --- |

④Are you in charge of the patients?

| 1．Yes | 2．No |
| --- | --- |

（２）About your working experience.

①Which license(s) do you have? (Multiple answers allowed)

| 1．Registered nurse | 2．Practical nurse | 3．Public Health nurse | 4．Care manager |
| --- | --- | --- | --- |

②How many years do you work as a nurse?

| 1．<4 | 2．5-9 | 3．10-14 | 4．15-19 |
| --- | --- | --- | --- |
| 5．20-24 | **6．25-29** | **7．30<** |  |

③How many years do you work as a home-visiting nurse?

| 1．<4 | 2．5-9 | 3．10-14 | 4．15-19 |
| --- | --- | --- | --- |
| 5．20-24 | **6．25-29** | **7．30<** |  |

④Do you have an experience of working at a hospital?

| 1．Yes | 2．No |
| --- | --- |

（３）About job condition.

①How many years do you work at the current agency?

| 1．<4 | 2．5-9 | 3．10-14 | 4．15-19 |
| --- | --- | --- | --- |
| 5．20-24 | **6．25-29** | **7．30<** |  |

②What is your employment status?

| 1．Full-time | 2．Part-time |
| --- | --- |

③What is your pay system?

| 1．Monthly | 2．Hourly | 3．Fixed by the number of home visiting |
| --- | --- | --- |

④How many patients’ homes do you visit for one week?

| 1．1-9 | 2．10-19 | 3．20-29 | 4．30-39 |
| --- | --- | --- | --- |
| 5．40-49 | 6．**50<** |  |  |

⑤Do you have a role of a care manager?

| 1．Yes | 2．No |
| --- | --- |

⑥Do you have a role of a director?

| 1．Yes | 2．No |
| --- | --- |

⑦How many days do you work on weekends and holidays for one month?

| 1．No | 2．1-2 | 3．3-4 | 4．5-6 | 5．7-8 |
| --- | --- | --- | --- | --- |

⑧How many days are you on call at night for one month?

| 1．No | 2．1-4 | 3．5-9 | 4．10-14 |
| --- | --- | --- | --- |
| 5．15-19 | 6．**20<** |  |  |

⑨Did you join a workshop in/out of the agency for the latest three months?

| 1．Always | 2．Often | 3．Rarely | 4．Never |
| --- | --- | --- | --- |

⑩Did you have a lunch with colleagues for the latest three months?

| 1．Always | 2．Often | 3．Rarely | 4．Never |
| --- | --- | --- | --- |

（４）About your health condition for the latest three months.

①How is your health condition?

| 1．Very good | 2．Good | 3．Acceptable | 4．Poor | 5．Very poor |
| --- | --- | --- | --- | --- |

②How is your sleep quality?

| 1．Good | 2．Fairly good | 3．Fairly poor | 4．Poor |
| --- | --- | --- | --- |

（５）About communication in the workplace.

①Do you have a friendly adviser in the agency?

| 1．Yes | 2．No |
| --- | --- |

②Do you have a friendly director in the agency?

| 1．Yes | 2．No | 3．I am a director. |
| --- | --- | --- |

③Do you use any communication tools to communicate with more than one nurse at the same time?（e.g. LINE, Skype, Mailing list）

| 1．Yes | 2．No |
| --- | --- |

（６）About patient information sharing.

Patient information was defined as the essential information including nursing daily reports on patient care for close monitoring and appropriate intervention in maintaining patient wellness.

(e.g. Patient’s condition reported by a home-visiting nurse colleague, physical information, living environment including family caregivers and utilization of medical care and social welfare services.)

①Can you visit a patient’s home with grasping patient’s information sufficiently?

| 1．Strongly agree | 2．Agree | 3．Disagree | 4．Strongly disagree |
| --- | --- | --- | --- |

（７）Opportunities or measures of patient information sharing in the workplace

①Do you use an electronic chart to share/grasp the patient information?

| 1．Always | 2．Often | 3．Rarely | 4．Never |
| --- | --- | --- | --- |

②Do you talk with your colleagues to share/grasp the patient information?

| 1．Always | 2．Often | 3．Rarely | 4．Never |
| --- | --- | --- | --- |

③Do you use an e-mail/SNS to share/grasp the patient information?

| 1．Always | 2．Often | 3．Rarely | 4．Never |
| --- | --- | --- | --- |

④Do you use a phone call to share/grasp the patient information?

| 1．Always | 2．Often | 3．Rarely | 4．Never |
| --- | --- | --- | --- |

⑤Do you join a face-to-face hand over to share/grasp the patient information?

| 1．Always | 2．Often | 3．Rarely | 4．Never |
| --- | --- | --- | --- |
| 5．Not available |  |  |  |

⑥Do you use a summary of nurse report to share/grasp the patient information?

| 1．Always | 2．Often | 3．Rarely | 4．Never |
| --- | --- | --- | --- |

⑦Do you attend a conference in the agency to share/grasp the patient information?

| 1．Always | 2．Often | 3．Rarely | 4．Never |
| --- | --- | --- | --- |
| 5．Not available |  |  |  |

（８）About job satisfaction

①Are you satisfied with your current work?

| 1．Strongly agree | 2．Agree | 3．Disagree | 4．Strongly disagree |
| --- | --- | --- | --- |

②Do you prefer your job as a home-visiting nurse?

| 1．Strongly agree | 2．Agree | 3．Disagree | 4．Strongly disagree |
| --- | --- | --- | --- |

③Do you think you are suitable for a home-visiting nurse?

| 1．Strongly agree | 2．Agree | 3．Disagree | 4．Strongly disagree |
| --- | --- | --- | --- |

④Do you feel rewarding for a home-visiting nursing care?

| 1．Strongly agree | 2．Agree | 3．Disagree | 4．Strongly disagree |
| --- | --- | --- | --- |

**Here is the end of the questionnaire.**

**Thank you for your cooperation.**
